# β-Hydroxybutyrate in Cardiovascular Diseases : A Minor Metabolite of Great Expectations

**DOI:** 10.3389/fmolb.2022.823602

**Published:** 2022-06-13

**Authors:** Shao Wei, Liu Binbin, Wu Yuan, Zhang Zhong, Lin Donghai, Huang Caihua

**Affiliations:** ^1^ Research and Communication Center of Exercise and Health, Xiamen University of Technology, Xiamen, China; ^2^ Xiamen Cardiovascular Hospital, Xiamen University, Xiamen, China; ^3^ Key Laboratory of Chemical Biology of Fujian Province, College of Chemistry and Chemical Engineering, Xiamen University, Xiamen, China

**Keywords:** cardiovascular diseases, cardiac energy metabolism, β-hydroxybutyrate, super fuel, signaling metabolite, cardiovascular therapies

## Abstract

Despite recent advances in therapies, cardiovascular diseases ( CVDs ) are still the leading cause of mortality worldwide. Previous studies have shown that metabolic perturbations in cardiac energy metabolism are closely associated with the progression of CVDs. As expected, metabolic interventions can be applied to alleviate metabolic impairments and, therefore, can be used to develop therapeutic strategies for CVDs. β-hydroxybutyrate (β-HB) was once known to be a harmful and toxic metabolite leading to ketoacidosis in diabetes. However, the minor metabolite is increasingly recognized as a multifunctional molecular marker in CVDs. Although the protective role of β-HB in cardiovascular disease is controversial, increasing evidence from experimental and clinical research has shown that β-HB can be a “super fuel” and a signaling metabolite with beneficial effects on vascular and cardiac dysfunction. The tremendous potential of β-HB in the treatment of CVDs has attracted many interests of researchers. This study reviews the research progress of β-HB in CVDs and aims to provide a theoretical basis for exploiting the potential of β-HB in cardiovascular therapies.

## Introduction

Cardiovascular diseases (CVDs) include a group of heart and blood vessel disorders, ranging from the peripheral artery, coronary artery, cardiac valve, cardiac muscle, and congenital heart diseases to arrhythmias and, ultimately, heart failure (Kalayinia et al., 2018) (Hajar, 2016). Despite the recent advances in clinical therapy, CVDs are still the leading cause of mortality worldwide (Kalayinia et al., 2021) (Chen et al., 2020). Hence, an in-depth molecular mechanistic understanding of CVDs is of great significance.

Recent studies reported that the primary mechanisms underlying the pathology of CVDs are closely relevant to metabolic perturbations (Yurista et al., 2021) (Ussher et al., 2016). As it is well-known, chronic obesity was a pathogenic factor of metabolic imbalance leading to CVDs (Lopaschuk et al., 2007). Obesity causes remarkable changes in cardiac energy metabolism, and the prominent effect is increasing the fatty acid uptake and oxidation by the heart (Lopaschuk et al., 2007). Moreover, fat accumulation can directly contribute to CVDs through reduced insulin sensitivity, impaired insulin production, and decreased glucose uptake in various tissues (Peters et al., 2017) (Wang et al., 2019) (Becker et al., 2017). Reactive oxygen species (ROS) were another potential pathogenic factor for CVDs, and the pathogenic mechanism is associated with metabolic disorders (Roth et al., 2017) (Geesala et al., 2020). As a by-product of cell respiration, ROS result from the metabolism of oxygen and are continuously produced in all aerobic organisms (Wang et al., 2020a). Increased ROS levels can lead to decreased availability of nitric oxide and vasoconstriction, subsequently promoting arterial hypertension (Senoner and Dichtl, 2019). ROS also negatively affect myocardial calcium treatment (Wang et al., 2020a), inducing arrhythmias and cardiac remodeling by facilitating hypertrophic signal transduction and apoptosis (Senoner and Dichtl, 2019). ROS also promote atherosclerotic plaque formation (Bertero and Maack, 2018). Even in the absence of obesity and dynamics of ROS, alterations in substrate metabolism of numerous organs resulting from the onset of CVDs can contribute to metabolic impairments in patients (Ussher et al., 2016). At the same time, CVDs can change the body and myocardial metabolism, usually accompanying the worsening of cardiac function and health outcomes (Ussher et al., 2016). From another perspective, the heart is the organ with the highest energy expenditure and oxidative demand (Bertero and Maack, 2018). The perturbations in cardiac energy metabolism, hence, could be significant contributors to CVDs.

As described earlier, exploring the effects of metabolic interventions on the improvement of cardiac dysfunction will provide a new direction for the treatments of CVDs. β-hydroxybutyrate, the most prominent ketone body, was once deemed to be a harmful and toxic substance, leading to ketoacidosis in patients with diabetes (Møller, 2020). Until now, increasing experimental and clinical research evidences have revealed the therapeutic potentials of β-HB in CVDs (Han et al., 2020). Traditionally, the concept of β-HB is the energy metabolic substrate, representing an alternative fuel source, for oxidative tissues, including the brain, heart, and skeletal muscle, in starvation and carbohydrate shortage (Evans et al., 2017; Monsalves-Alvarez et al., 2020; Wang et al., 2021). Moreover, β-HB is widely linked to various cellular processes by regulating gene transcription (Han et al., 2018), inflammation and oxidative stress (Wang et al., 2020a), cardiac remodeling (Sultan, 1990), and cardiovascular risk factors (Cotter et al., 2013). The roles played by both the metabolic substrate and signal molecule potentially allow β-HB to be used to treat cardiac dysfunctions and other vascular diseases. This study reviews the research progress of the metabolic and signaling effects of β-HB and provides a theoretical basis for exploiting the potentials of β-HB in CVDs therapies.

## The Biological Synthesis and Utilization of β -Hydroxybutyrate

β-HB, a chiral molecule with two enantiomers (R/D and S/L), is the most abundant ketone body in mammals, significantly contributing to the dynamic range of ketone body levels (Evans et al., 2017) (Takahashi et al., 2019). The synthesis of β-HB occurs mainly in the liver mitochondrial matrix with a series of enzymes (Mierziak et al., 2021). BDH1, a core enzyme, which catalyzes the final step in the β-HB synthesis, can introduce chiral specificity (Lincoln et al., 1987). Due to the BDH-induced chiral specificity, only R-3-β-HB is produced by normal metabolism and then is readily catabolized into acetyl-CoA and ATP (Newman and Verdin, 2017). Fasting, exercise, caloric restriction, ketogenic diet, and other approaches resulting in endogenous production of β-HB can produce R-3-β-HB rather than S-3-β-HB (Mierziak et al., 2021) (Newman and Verdin, 2017). Thus, β-HB in this review article is mainly referring to R-3-β-HB.

**FIGURE 1:**
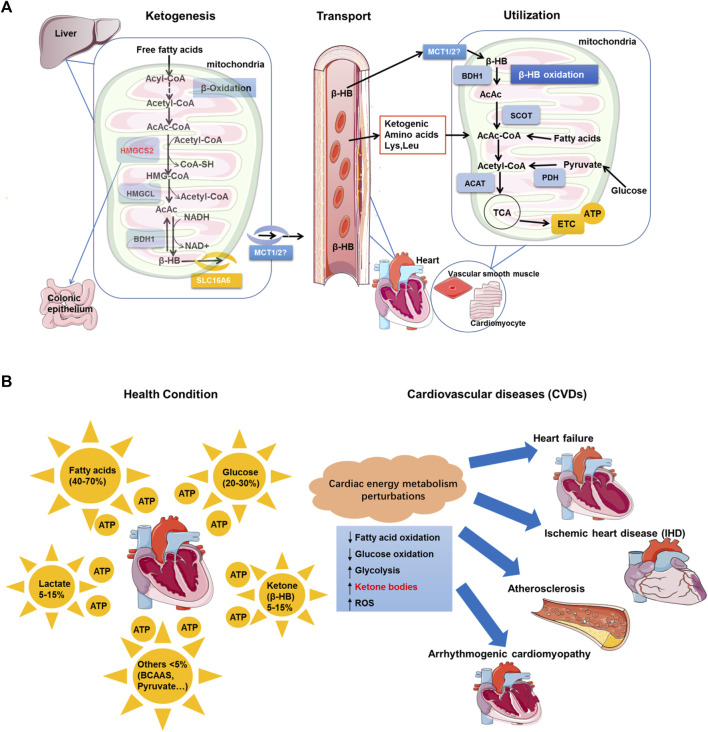
Perturbations of cardiac energy metabolism in CVDs and β-HB utilization in cardiomyocytes. **(A).** Biological synthesis of β-HB mainly occurs in hepatic mitochondria, where the fate-committing enzyme HMGCS2 is expressed. β-HB is synthesized from acetyl-CoA that is derived from β-oxidation. β-HB is transported through the circulatory system. After being transported, β-HB can be oxidized in extrahepatic organs, including the heart and vascular smooth muscle. Most extrahepatic mitochondria lack HMGCS2, while they have abundant enzymes for β-HB utilization, including BDH1, SCOT, and ACAT. In cardiomyocytes, β-HB can generate acetyl-CoA to enter the TCA cycle and electron transport chain (ETC), finally producing ATP. **(B)**. In healthy conditions, fatty acids, glucose, lactate, ketone bodies (principally, β-HB), and other metabolites are substrates for cardiac energy metabolism. The perturbations in cardiac energy metabolism are significant contributors to cardiovascular pathologies, including heart failure, ischemic heart disease (IHD), atherosclerosis, and arrhythmogenic cardiomyopathy. In the case of CVDs, the myocardial fatty acid oxidation rates and glucose oxidation alterations are decreased. On the opposite, the level of myocardial glycolysis, ketone bodies, and ROS is observably increased.

As is well known, the synthesis of β-HB (Figure 1A) begins with the condensation of two acetyl-CoA molecules to form acetoacetyl-CoA in a reaction catalyzed by beta-ketothiolase (Mierziak et al., 2021). The well-established regulation of β-HB synthesis primarily depends on the substrate availability in the form of fatty acids and the expression and activity of the enzyme HMG-CoA synthase (HMGCS2; EC 2.3.3.10) (Newman and Verdin, 2017) (Garber et al., 1974). Robust expression of HMGCS2 is restricted to hepatocytes and colonic epithelium (Chen et al., 2017). The HMGCS2 enzyme catalyzes a fate-committing ketogenic reaction: condensation of β-oxidation–derived acetoacetyl-CoA (AcAc-CoA) and acetyl-CoA to generate HMG-CoA, which is then cleaved by HMGCL to generate acetoacetic acid (AcAc) (Puchalska and Crawford, 2017). AcAc is reduced to β-HB in an NAD-/NADH-coupled near-equilibrium reaction, which is catalyzed by phosphatidylcholine-dependent mitochondrial BDH1 (Cotter et al., 2013). BDH1 modulates mitochondrial redox in the liver and extrahepatic tissues, in which the ratio of AcAc to β-HB is directly proportional to that of mitochondrial NAD + to NADH (Williamson et al., 1967). However, molecular mechanisms precisely controlling the ketogenic rate remain to be addressed in detail (Puchalska and Crawford, 2017).

Although the synthesis of β-HB occurs mainly in the liver, its utilization occurs mainly in extrahepatic tissues (Cotter et al., 2014a) (Cotter et al., 2014b). That is because liver cells have a strong enzyme system for synthesizing β-HB but lack the enzyme systems for utilizing β-HB (Puchalska and Crawford, 2017). Nonetheless, the extrahepatic tissues, such as the brain, myocardium, and skeletal muscle have abundant and efficient ketone body–decomposing enzymes, which can break down ketone bodies to regenerate acetyl CoA. Thereafter, acetyl CoA is oxidized for supplying energy (Williamson et al., 1967). In addition, as a polar molecule, β-HB is readily soluble in water and blood. In the blood, β-HB can be transported to extrahepatic tissues, where they primarily undergo terminal oxidation to contribute to the TCA cycle (Cotter et al., 2013). The circulating β-HB level is always related to physiological and pathological conditions. In general, the β-HB level greater than 0.5 mM has been considered a cut-off point for entry into ketosis (Chu et al., 2021). In pathological conditions, such as diabetes, the serum β-HB level can be elevated to as high as 20 mM. In neonates, however, upon fasting, prolonged exercise, or following a ketogenic diet, the serum β-HB level can be increased up to 1–8 mM (Adal et al., 2006) (Harvey et al., 2019). It was previously reported that the β-HB levels in patients with congestive heart failure (CHF) were increased to about 2.67 mM, and the increase of β-HB was in proportion to the severity of cardiac dysfunction (Lommi et al., 1996). Although the increased circulating β-hydroxybutyrate has been reported in a variety of CVDs since the 1990s (Lommi et al., 1996), it took a long time to clarify the mechanism behind this phenomenon.

Previous studies have shown that monocarboxylic acid transporters (MCTs) can transport β-HB out of the mitochondrial membrane and liver cell plasma membrane (Puchalska and Crawford, 2017) (Abdul Kadir et al., 2020). However, the transport of β-HB is less understood, relative to the synthesis and utilization of β-HB. The monocarboxylate transporter SLC16A6 has been identified to be a key transporter for exporting β-HB from the liver (Hugo et al., 2012). Many transporters are present in the cells; however, a small number of them have been characterized. Whether other transporters facilitate either the uptake of β-HB into target tissues or its intracellular movement needs further research. β-HB can also be transported into cardiomyocytes *via* monocarboxylate transporter 1 (MCT1) and MCT2 and then enter the mitochondria (Puchalska and Crawford, 2017). The uptake and utilization of ketone bodies by cardiomyocytes is a complex process of synergistic action of multiple enzymes (Abdul Kadir et al., 2020), and we will cover this in detail in the following sections.

## β -Hydroxybutyrate in Cardiac Energy Metabolism and its Metabolic Effects on Cardiovascular Disease

Given that the heart is the organ with the highest energy expenditure and oxidative demand (Chandler et al., 2004), it can be expected that perturbations in cardiac energy metabolism are significant contributors to the progression of CVDs (Ussher et al., 2016) (Song et al., 2020). In addition to glucose and fatty acids, the heart also oxidizes various substrates, including β-HB, lactate, and amino acids (Kolwicz et al., 2016). Moreover, it has been demonstrated that β-HB can serve as an energy source in the absence of sufficient blood glucose, which is of particular importance during starvation or illness (Puchalska and Crawford, 2017). Thus, the effects of β-hydroxybutyrate on cardiac energy metabolism and cardiovascular disease have interested many researchers.

Cardiac energy metabolism has been well-documented (Lopaschuk et al., 2007) (Abdul Kadir et al., 2020) (Abozguia et al., 2009) (Figure 1B). In healthy conditions, metabolic flexibility is a crucial feature of cardiac energy metabolism (Maack et al., 2018). A healthy heart can derive energy from various circulating substrates, including fatty acids, glucose, amino acids, ketone bodies, and lactate. Fatty acid metabolites contribute to the main ATP production of the heart (>60%) (Qian and Wang, 2020). Interestingly, compared to fatty acids, glucose is less consumed (Lopaschuk et al., 2007) (Murashige et al., 2020). Glycolysis is responsible for only about 5% of the ATP production in the normal oxygenated heart (Abozguia et al., 2009), although low availability, lactate, ketone bodies (prominent, β-HB), and amino acids also contribute to the ATP production of the heart (Abozguia et al., 2009). As indicated by the previous works, the myocardium displays the highest β-HB consumption and oxidizes ketone bodies in proportion to prevailing concentrations at the cost of glucose and fatty acids (Veech, 2004). Furthermore, the metabolic flexibility of a healthy heart is highly dynamic, as demonstrated by its ability to rapidly change the pattern of fuel utilization to adapt to the substrate and hormonal environment (Ussher et al., 2016).

On the contrary, the progression of CVDs is associated with loss of metabolic flexibility of cardiomyocytes (Yurista et al., 2021). The energy deficit is common in cardiomyocytes, and these metabolic alterations depend on the stages of disease pathophysiology (Chandler et al., 2004). Even in the early stages, the transition of energy substrate utilization from fatty acids to glucose also occurs in the structural heart disease (Sack et al., 1996). Moreover, the altered utilization of the energy substrate also plays a key role in the progression of heart failure (HF) (Maack et al., 2018) (Stanley et al., 2005). Intriguingly, in the context of reduced fatty acid oxidation, the failing heart appears to reprogram metabolism to increase reliance on ketone bodies acting as a fuel source (Aubert et al., 2016; Bedi et al., 2016; Horton et al., 2019). In the pathologically remodeled heart (e.g., hypertension or myocardial infarction) and the diabetic heart, the O_2_ consumed for ATP production during ketone metabolism is more efficient than FAO (Bertero and Maack, 2018). Compared to FAO, ketone body oxidation is energetically efficient, yielding more energy for ATP synthesis per molecule of oxygen invested (the ratio of phosphate/oxygen [P/O]) (Sato et al., 1995) (Kashiwaya et al., 2010) (Veech, 2004). As is known, ischemic heart disease (IHD) remains the leading cause of cardiovascular death globally (GBD 2017 Causes of Death Collaborators, 2015). The myocardial intermediary energy metabolism is significantly altered in IHD (Ussher et al., 2016). In response to the downregulated oxidative metabolism, glycogen breakdown and glycolysis rates are increased, as glycolysis can produce ATP anerobically rapidly (Taegtmeyer et al., 2016). Glucose oxidation rates are also markedly decreased in the ischemic myocardium, whereas glycolytic rates are significantly increased due to the stimulation of glycogenolysis (Wisneski et al., 1987) (Vary et al., 1981). On the other hand, several works reported minimal reliance of a healthy heart on amino acids acting as a source of ATP (Monsalves-Alvarez et al., 2020). Regardless of this, it has been suggested that amino acid metabolism in the heart may also be vital during ischemia (Drake et al., 2012). Atherosclerosis also represents another common form of CVDs. The presence of atherosclerotic plaques in the coronary vessels can trigger a vast majority of IHD. However, whether atherosclerosis can cause changes in myocardial energy metabolism, in general, remains poorly understood (Ussher et al., 2016).

The ketogenic shift seems to be a universal cardiac response to stress. β-HB acting as a fuel is particularly significant in the hypertrophied and failing heart (Birkenfeld et al., 2019). Moreover, in the context of ischemia or reperfusion injury, β-HB also confers potential cardioprotective effects (Wang et al., 2008) (Al-Zaid et al., 2007), possibly due to either the increased mitochondrial abundance in the heart or the upregulation of crucial oxidative phosphorylation mediators (Snorek et al., 2012). It is reported that plasma β-HB and its cardiac utilization increased in patients with arrhythmogenic cardiomyopathy (Song et al., 2020). A series of CVDs with heart failure increase the reliance on ketone bodies for cardiac ATP production and are accompanied by increased circulating β-HB levels in the blood (Lommi et al., 1996) (Abdul Kadir et al., 2020). Previous studies reveal that β-HB competes with other substrates in the heart as the fuel when the availability of fatty acid and carbohydrate is limited (Kolwicz et al., 2016; Abdul Kadir et al., 2020). In the animal model, studies indicate that fatty acid utilization is downregulated and ketone body utilization is upregulated in failing hearts of mice (Aubert et al., 2016). Thus, the shift of energy metabolism to ketone body metabolism is an efficient alternative avenue for oxidative ATP production in CVDs.

Although preliminary interventional and observational studies indicate potential benefits of the metabolic effects of β-HB in the heart, an adverse viewpoint still exists. In cardiomyocytes, an early study reported that β-HB causes concurrent inhibition of glucose metabolism and FAO metabolism, thereby impairing myocardial energy supply (Taegtmeyer, 1994). Nevertheless, recent studies directly measured cardiac β-HB, glucose, and FAO metabolism in mouse models, and the findings are not consistent with those of the previous study (McCommis et al., 2020) (Brahma et al., 2020). Furthermore, increased β-HB levels do not always correlate with positive clinical outcomes in humans. Arrhythmogenic cardiomyopathy (AC) is a severe disease that may cause sudden death, lacking clinical biomarkers. A recent study suggested that the elevated plasma level of β-HB might be a potential predictor of major adverse cardiovascular events (MACEs) and disease progression in patients with AC and their clinically asymptomatic relatives (Song et al., 2020). It was also reported that compared with normal controls, the atrial samples from patients with atrial fibrillation exhibited increased levels of ketone bodies (Xie et al., 2016). The metabolic effects of the increased level of circulating β-HB are not fully understood yet, and more research needs to be performed.

## Signaling Effects of β -Hydroxybutyrate on Cardiovascular Disease

**FIGURE 2:**
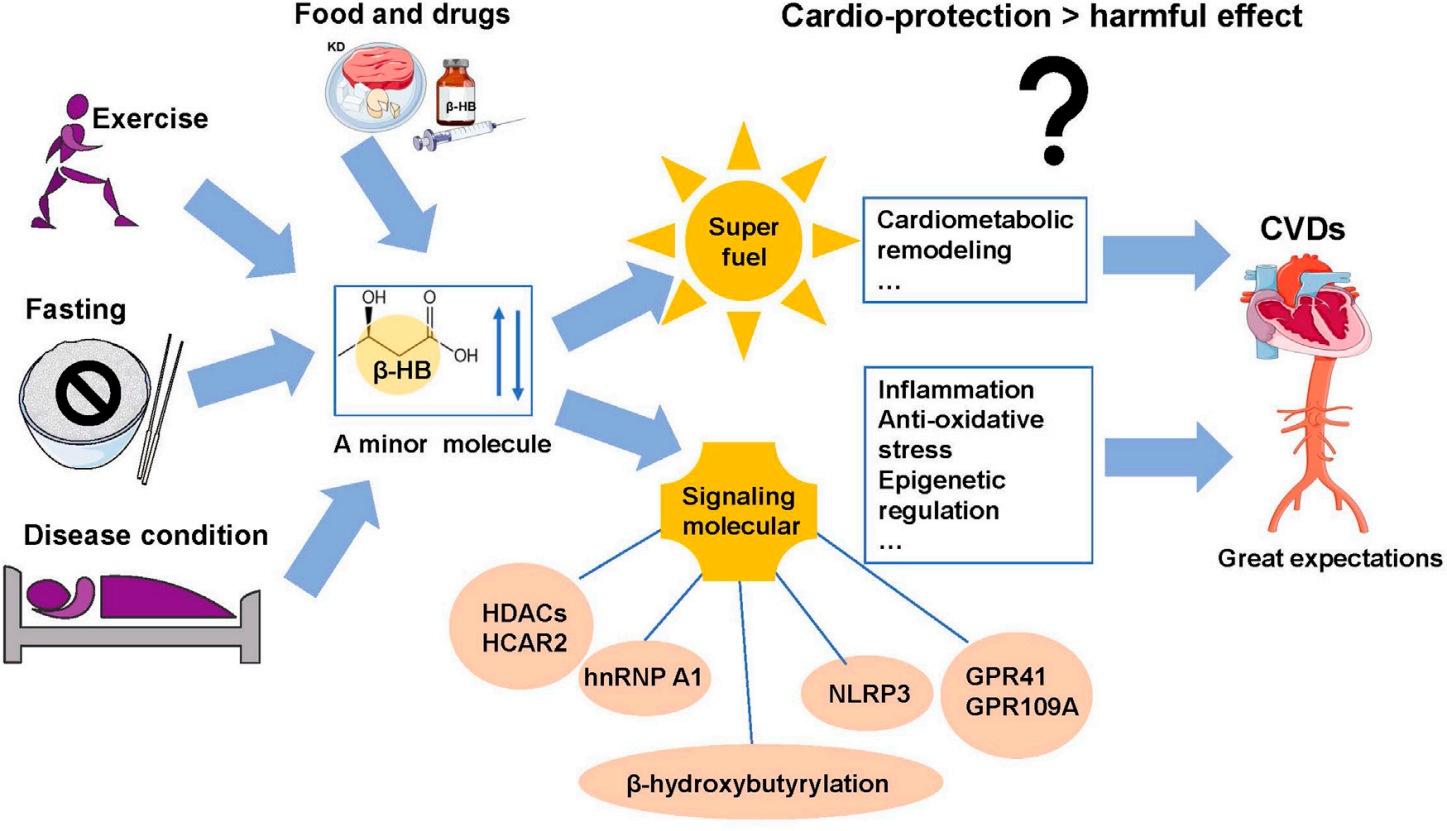
Summary of regulation of β-HB levels and its multi-effects on CVDs. Fasting, exercise, ketogenic diet, drugs, and some disease conditions change the endogenous β-HB levels. Beyond their contribution to energy generation, β-HB may exert signaling effects on inflammation, oxidative stress, cell death, and cardiac remodeling that may induce either the prospective cardiovascular protection or harmful effect on CVDs.

In addition to the metabolic effects, β-HB is also a signaling metabolite that regulates cellular signals by targeting diverse biomolecules (Newman and Verdin, 2017) (Puchalska and Crawford, 2017). The primary signaling actions of β-HB (Figure 2) are closely related to cardiovascular diseases, including binding to cell-surface receptors; inhibiting histone deacetylases (HADCs); acting as a substrate for protein posttranslational modification; and modulating potassium flux across the plasma membrane (Mierziak et al., 2021) (Newman and Verdin, 2017).

As is known, β-HB serves as an endogenous ligand for G protein–coupled receptors (GPRs) (Chu et al., 2021). β-HB can specifically bind to HCAR2 and activate HCAR2 to inhibit lipolysis of adipocytes, which might represent a feedback mechanism for regulating the availability of the fatty acid precursors of ketone body metabolism (Offermanns et al., 2011) (Taggart et al., 2005). To be more specific, HCAR2 activation in neurons can potentiate glutaminergic signaling that helps regulate blood pressure and heart rate (Rezq and Abdel-Rahman, 2016). In addition, β-HB also functions as a ligand for FFAR3 (also known as GPR41) which is another G protein–coupled receptor highly expressed in sympathetic ganglions throughout the body of mice (Nøhr et al., 2015) (Kimura et al., 2011). It was reported that β-HB can suppress sympathetic tone and heart rate through antagonistic action against FFAR3 during fasting (Oshima et al., 2019). Interestingly, it was also reported that β-HB acts as an agonist of FFAR3 and regulates voltage-dependent calcium channels (Won et al., 2013). Further works are required to confirm the regulatory effects of β-HB on FFAR3 functions. Furthermore, it is noteworthy that heterogeneous nuclear ribonucleoprotein A1 (hnRNP A1) was a new direct binding target of β-HB. Through hnRNP A1–mediated upregulation of Oct4, β-HB can prevent vascular senescence (Han et al., 2018).

β-HB also plays essential signaling roles in CVDs *via* inhibiting histone deacetylases (HADCs). HDACs play essential roles in regulating mitochondrial metabolism and function by balancing the acetylation activities of histone acetyltransferases (HATs) (Felisbino and McKinsey, 2018). It was previously reported that in many cardiac pathological conditions, such as heart failure, diabetic heart, and myocardial I/R injury, HDAC activities are significantly elevated (Granger et al., 2008; Aune et al., 2014). Inhibition of HDAC activities, hence, is an effective treatment for cardiac I/R injury and failing heart. As an endogenous inhibitor of Class I HDACs, β-HB possesses significant cardioprotective effects (Hasselbalch et al., 1996). By inhibiting HDAC activities, β-HB upregulates expressions of the Foxo3a and MT2 genes to reduce oxidative stress (Shimazu et al., 2013). In addition, β-HB also has inhibitory activity against HDAC1, which may be related to cardiomyocyte autophagy (Cao et al., 2011; Shimazu et al., 2013). However, whether the cardioprotective effects of β-HB are mediated by autophagy is still unclear. When β-HB was used to inhibit HDAC6, it did not show positive benefit and was even detrimental to cardiomyocytes during I/R injury (Aune et al., 2014). Furthermore, β-HB accumulation induced by a long-term ketogenic diet can inhibit HDAC2 and activate Sirt7 transcription, which is harmful to heart health by promoting cardiac fibrosis (Xu et al., 2021). Given the controversial results in cardio-protection, the complicated relationship between β-HB and HDACs remains to be addressed in detail.

On the other hand , the signaling roles of β-HB in the CVDs is linked to the inflammation. As is known, the inflammatory mechanisms in CVDs are closely associated with NLRP3, which has a vital role in innate immunity and inflammation (Wang et al., 2020b). Significantly, β-HB can function as an endogenous inhibitor of the NLRP3 inflammasome, attenuating inflammatory responses (Yamanashi et al., 2017). It was revealed that inhibition of potassium efflux contributes to the mechanism of β-HB, inhibiting the activation of the NLRP3 inflammasome (Kimura et al., 2011). Mice deficient in NLRP3 are protected from obesity and insulin resistance when fed on a high-fat diet (Yudkoff et al., 2007). The ablation of NLRP3 also has attenuated diabetes and atherosclerosis (Youm et al., 2015) (Duewell et al., 2010). Increasing the circulating β-HB levels can reduce the activation of the cardiac NLRP3 inflammasome in mice with heart failure (Byrne et al., 2020).

In addition, the signaling roles of β-HB in the CVDs also involved antioxidative stress and epigenetic regulation. ROS has negative effects on myocardial calcium treatment, inducing arrhythmias and cardiac remodeling by facilitating hypertrophic signal transduction and apoptosis (Senoner and Dichtl, 2019) (Huynh and Heo, 2019). Oxidation of ketone bodies may also curtail ROS production (Paoli, 2014). Administration of β-HB in mice can prevent liver ROS (Michal et al., 2015). Interestingly, under hypoxia conditions, reducing the serum level of β-HB can improve the excitation–contraction of cardiac cells (Klos et al., 2019). In addition, metabolism-mediated epigenetic changes represent an adapted mechanism for cellular signaling, which has been the historical focus of interest. β-HB can dramatically increase lysine β-hydroxybutyrylation of histone tails, which is an epigenetic marker associated with fasting responses and muscle catabolic states (Cavallucci and Pani, 2021). However, the regulatory mechanism underlying the β-hydroxybutyrate–mediated epigenetic pathway remains unclear, hindering its clinical functional research. Further studies need to be performed. In addition to the several signal roles of β-HB mentioned previously, it is reported that increases in circulating β-HB can mediate CVDs benefiting from the drugs of sodium-glucose cotransporter inhibitors (SGLT2i) (Packer, 2020). However, the mechanisms underlying the cardiovascular benefits of SGLT2i remain elusive, which might be related to the signaling role of β-HB (Kaplan et al., 2018).

### Future Perspectives

As summarized earlier, both the roles of the β-HB metabolism and the underlying mechanism are of great significance for the mechanistic understanding of the occurrence and development of pathological cardiac remodeling and for the guidance of clinical treatment. The minor metabolite, β-HB, has both advantages and disadvantages in CVDs. The current research reveals that the advantages of β-HB far outweigh the disadvantages (Sato et al., 1995) (Xu et al., 2021) (Nielsen et al., 2019) (Gormsen et al., 2017). Even though evidence for the beneficial effects of β-HB on cardiovascular disease is rapidly emerging, it is still unclear whether the role of β-HB is essentially adaptive or maladaptive in CVD_S_. In addition, it is also unclear what makes β-HB a double-edged sword in treating cardiovascular disease. Both the optimal timing and application strategy of β-HB for CVDs treatments are worthy of further exploration.

## Abbreviations

AcAc, acetoacetic acid; AC, arrhythmogenic cardiomyopathy; ACAT, acyl-coenzyme a-cholesterol acyltransferase; BDH, beta-hydroxybutyrate dehydrogenases; CVDs, cardiovascular diseases; ETC, electron transport chain; FAO, fatty acid oxidation; FFAR3, free fatty acid receptors 3; GPRs, G protein–coupled receptors; HDAC, histone deacetylase; HF, heart failure; I/R, ischemia/reperfusion; IHD, ischemic heart disease; KD, ketogenic diet; MACEs, major adverse cardiovascular events; MCT, monocarboxylate transporters; NLRP3, nucleotide-binding domain-like receptor protein 3; ROS, reactive oxygen species; SCOT, succinyl-CoA:3-ketoacid-CoA transferase; SGLT2i, sodium–glucose co-transporter 2 inhibitors; β-HB, beta-hydroxybutyrate.
